# Supplementary figures and images for: α-Ketoglutarate-Mediated DNA Demethylation Sustains T-Acute Lymphoblastic Leukemia upon TCA Cycle Targeting
Source: Cancers (Basel). 2022 Jun 16;14(12):2983. doi: 10.3390/cancers14122983 (PMC9221025; doi:10.3390/cancers14122983)

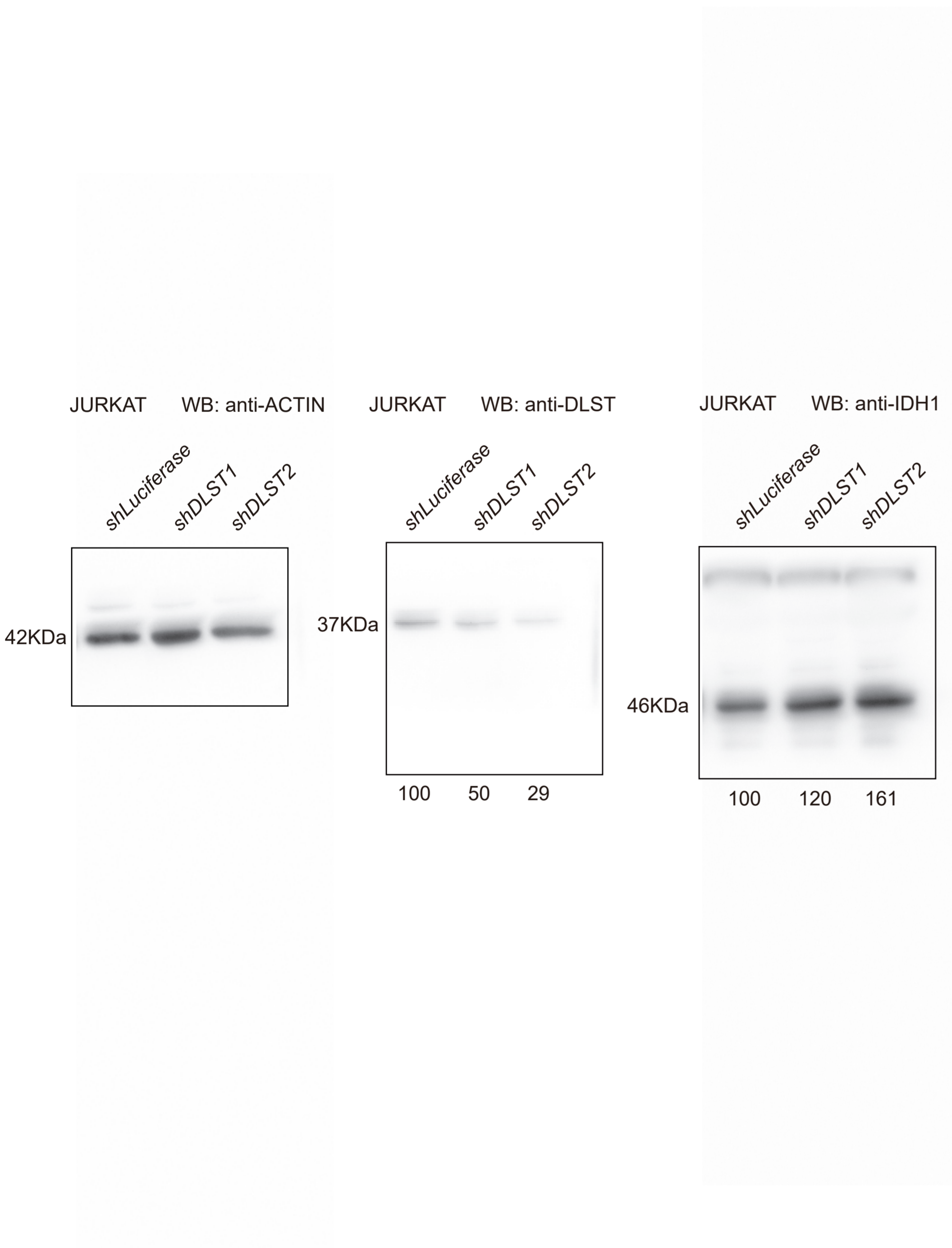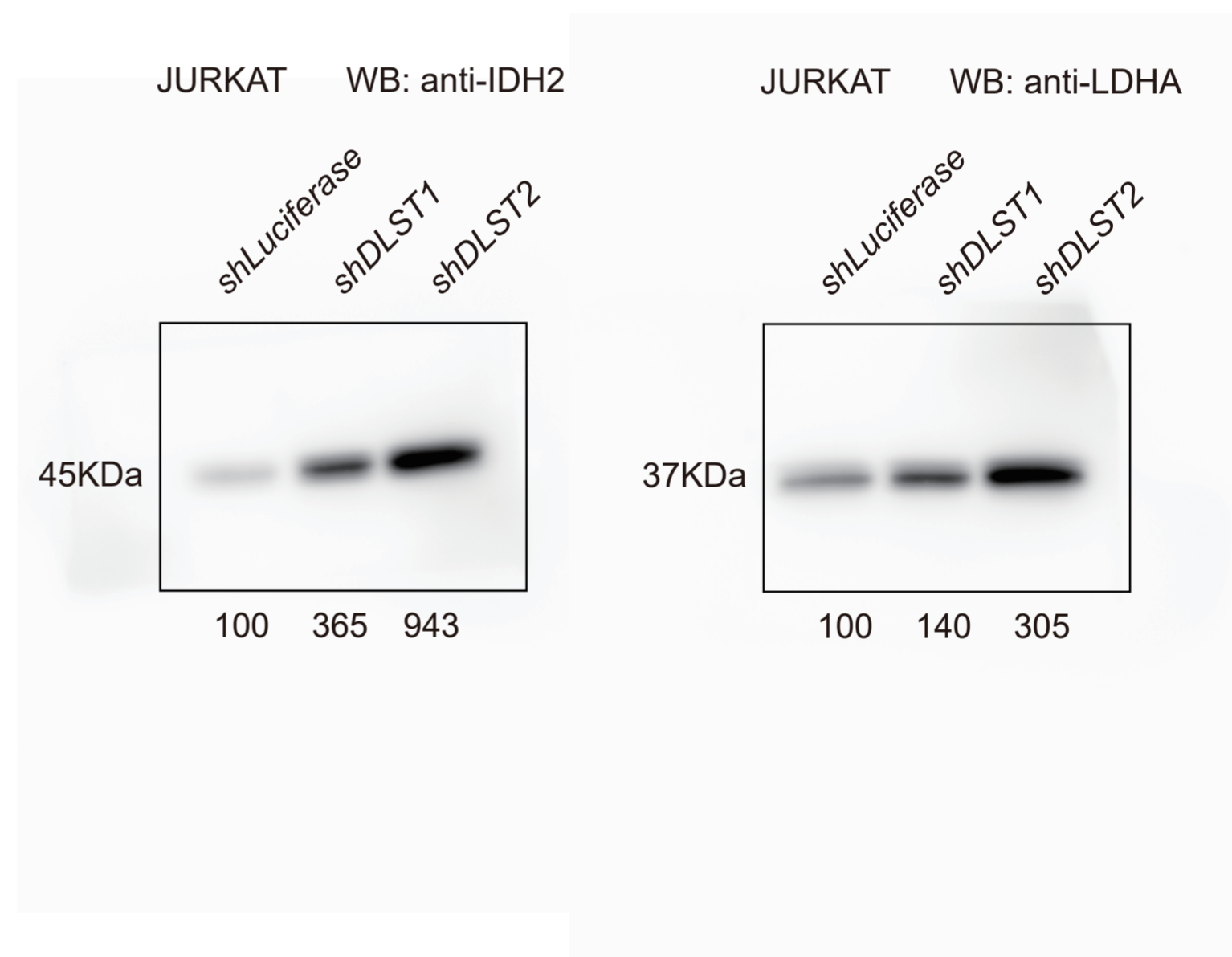

Supplement: Supplementary file 1 [file cancers-14-02983-s001.zip › Figure S1/Fig2 jurkat.pdf]

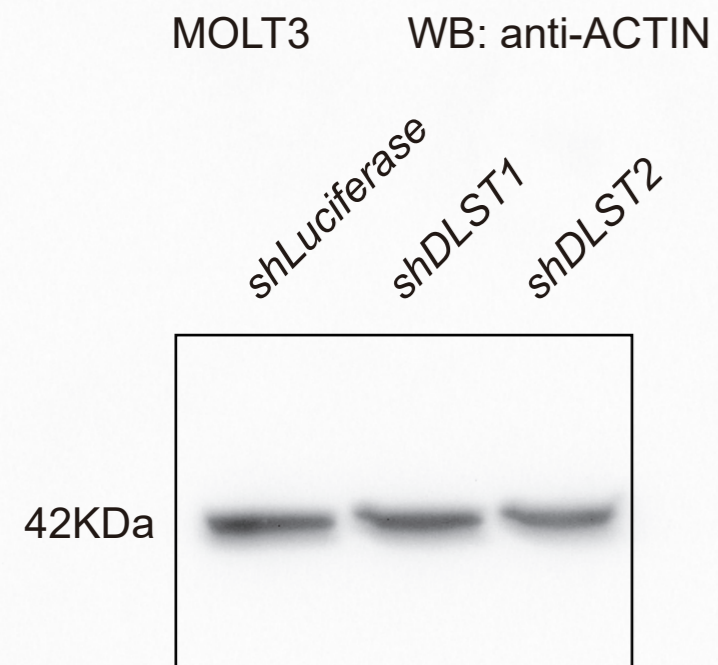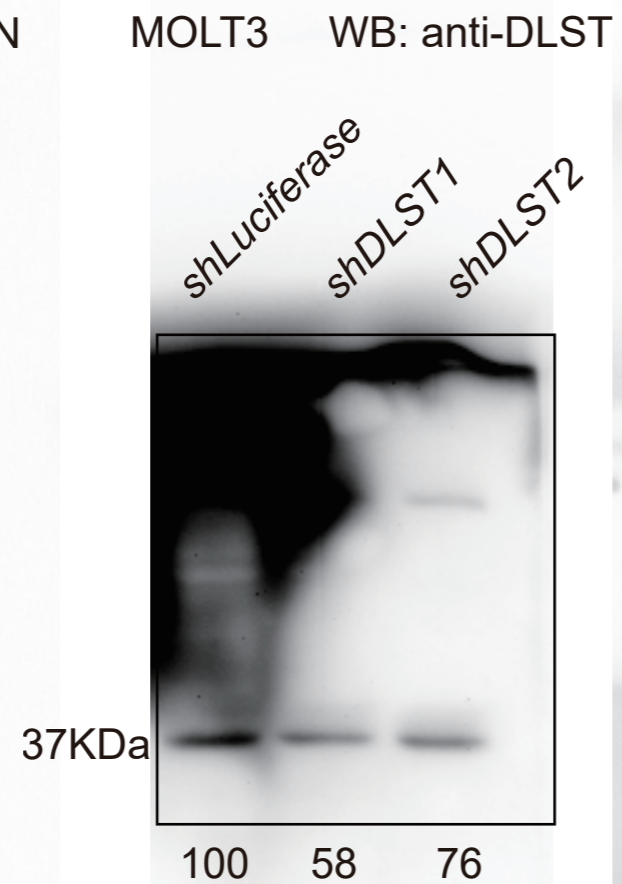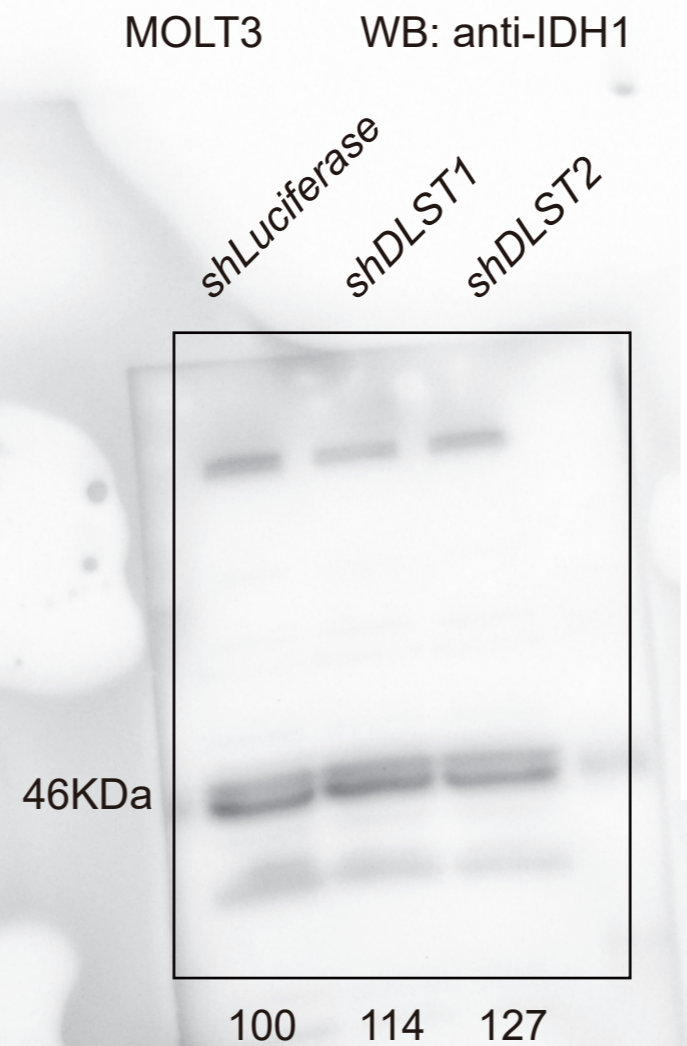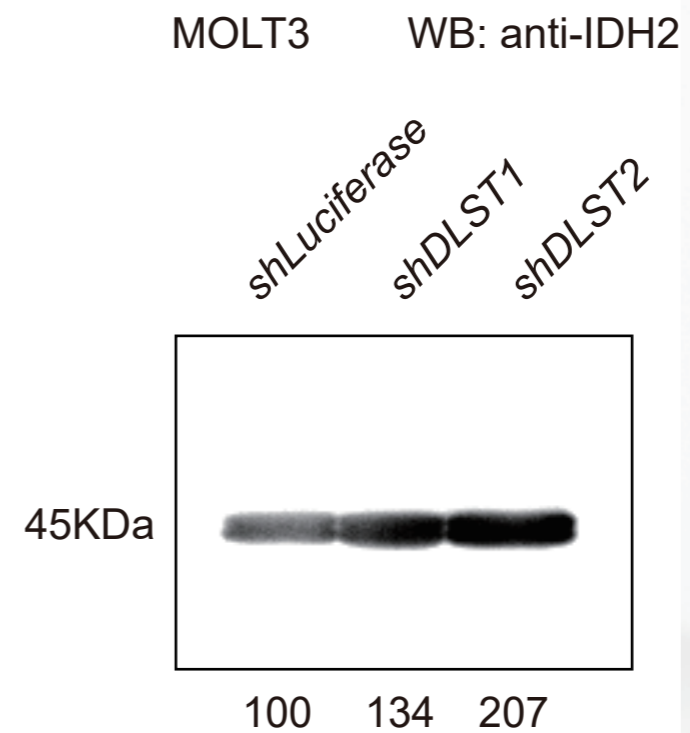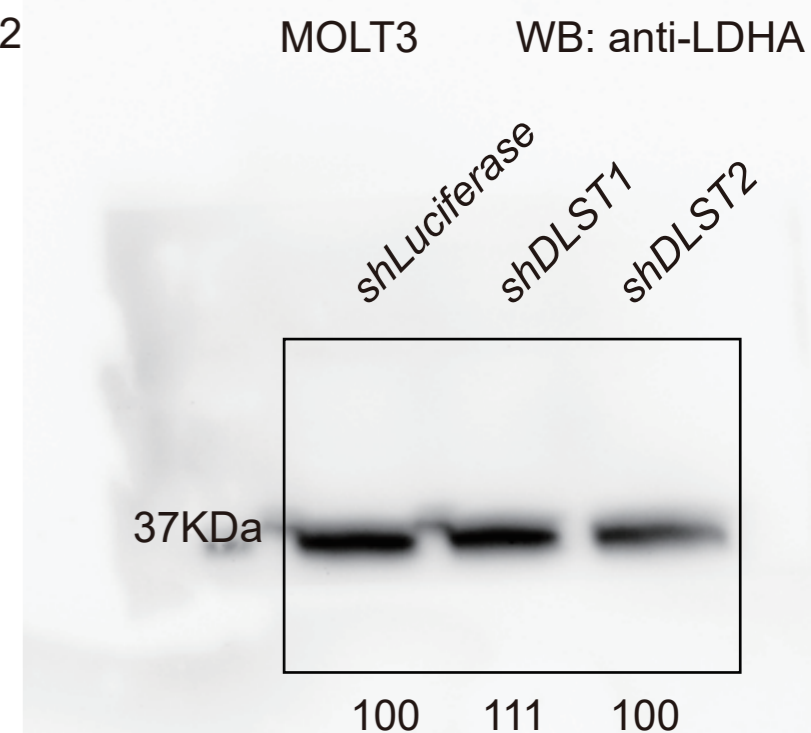

Supplement: Supplementary file 1 [file cancers-14-02983-s001.zip › Figure S1/Fig2 molt3.pdf]

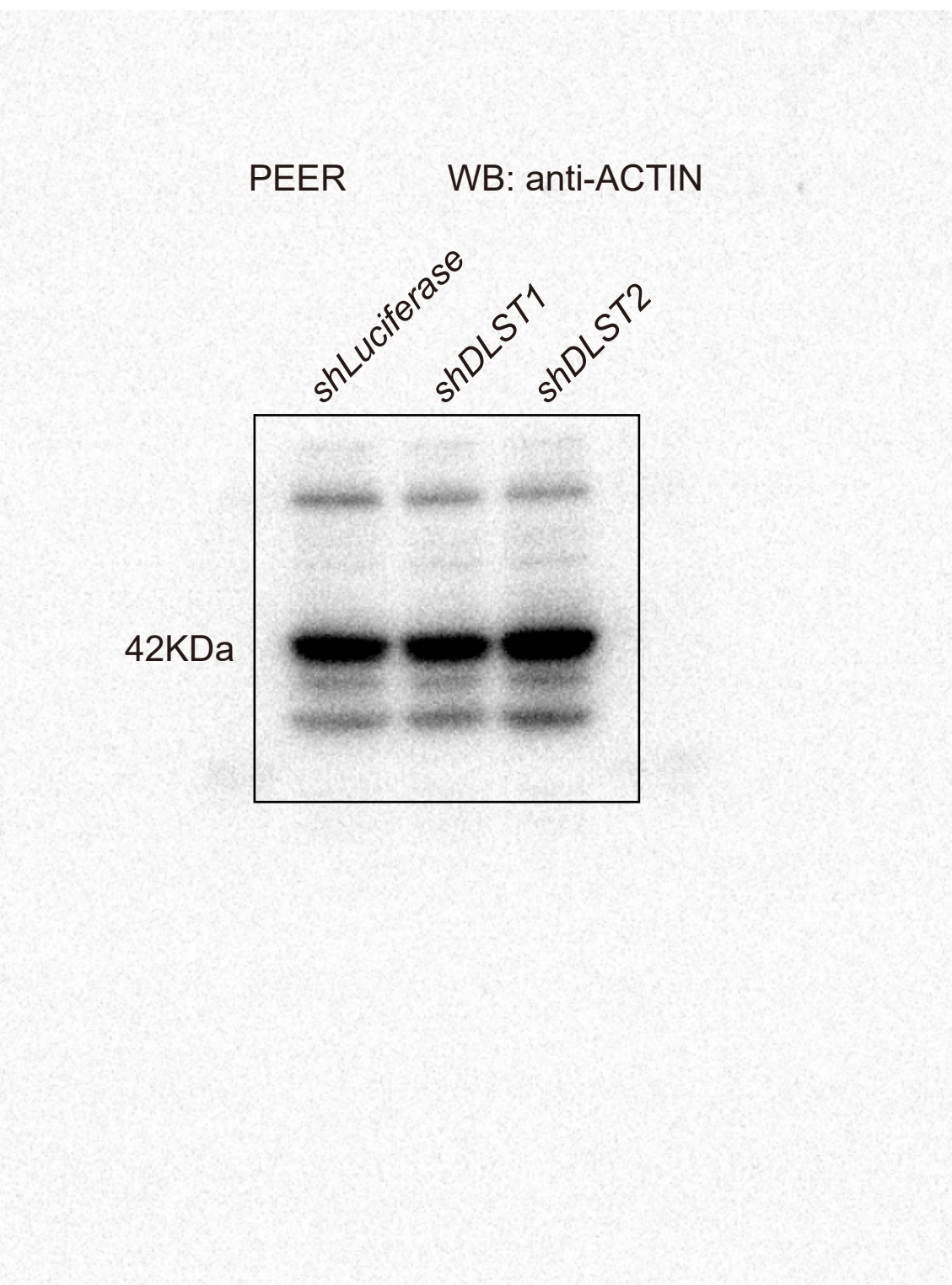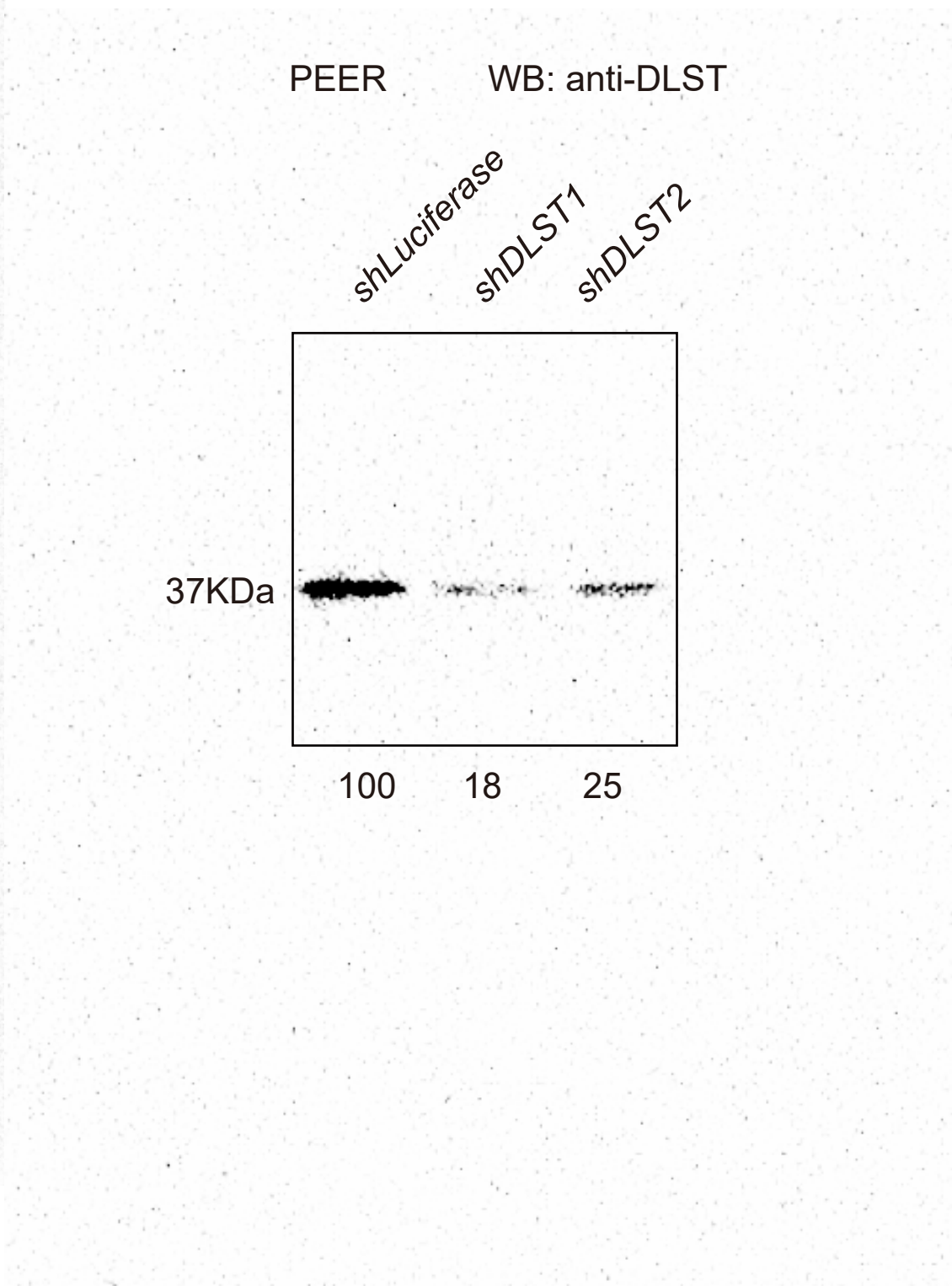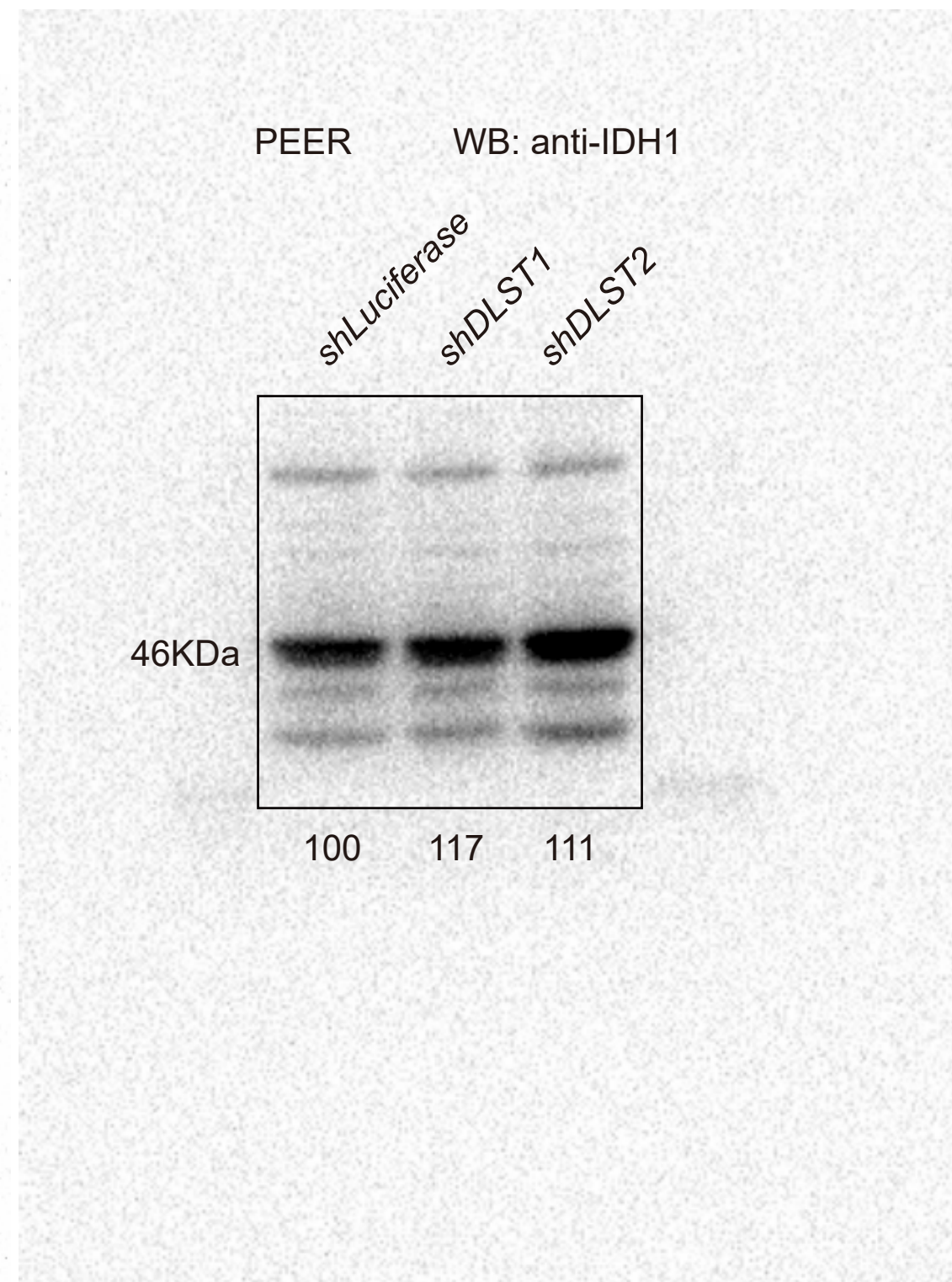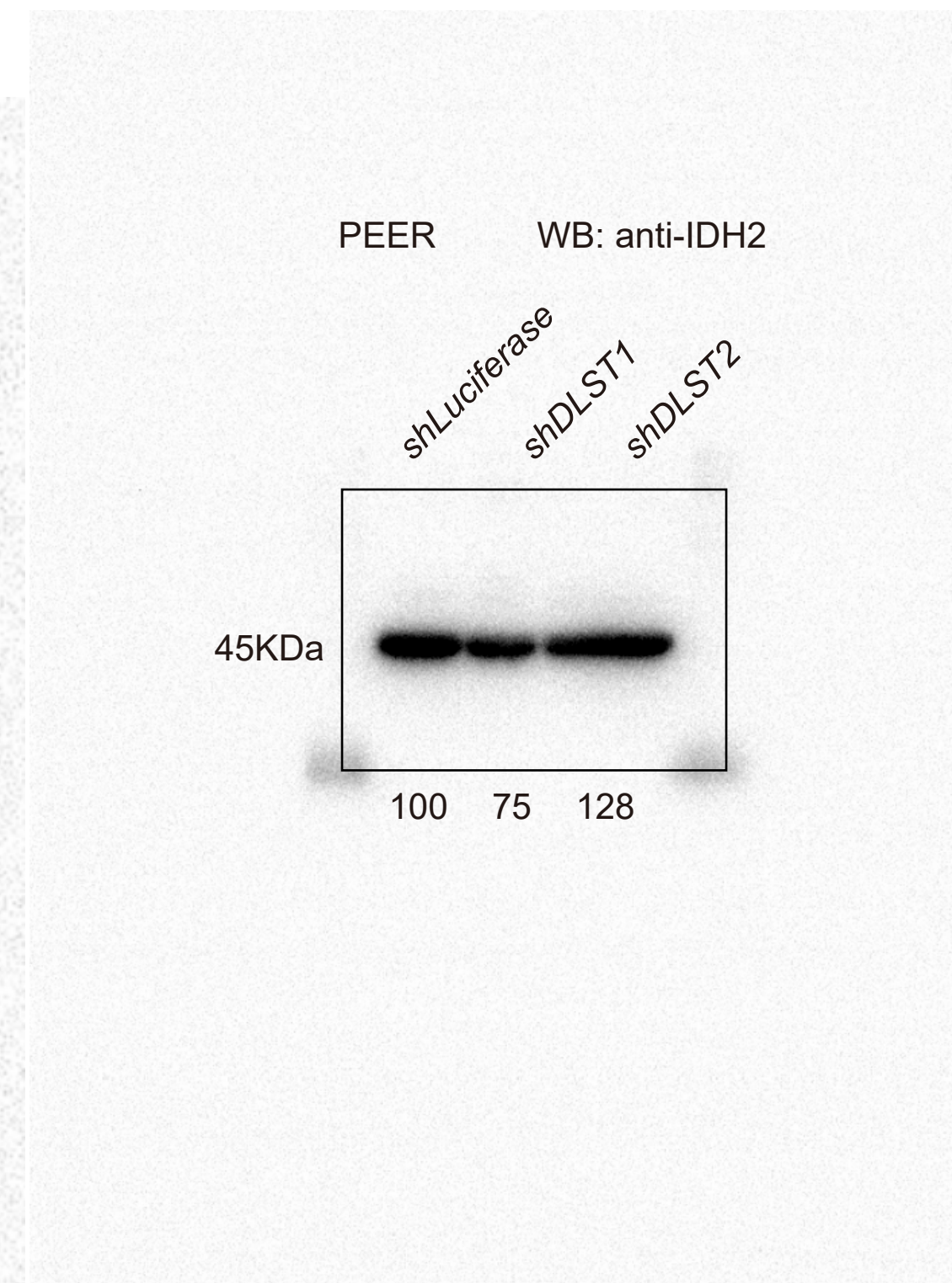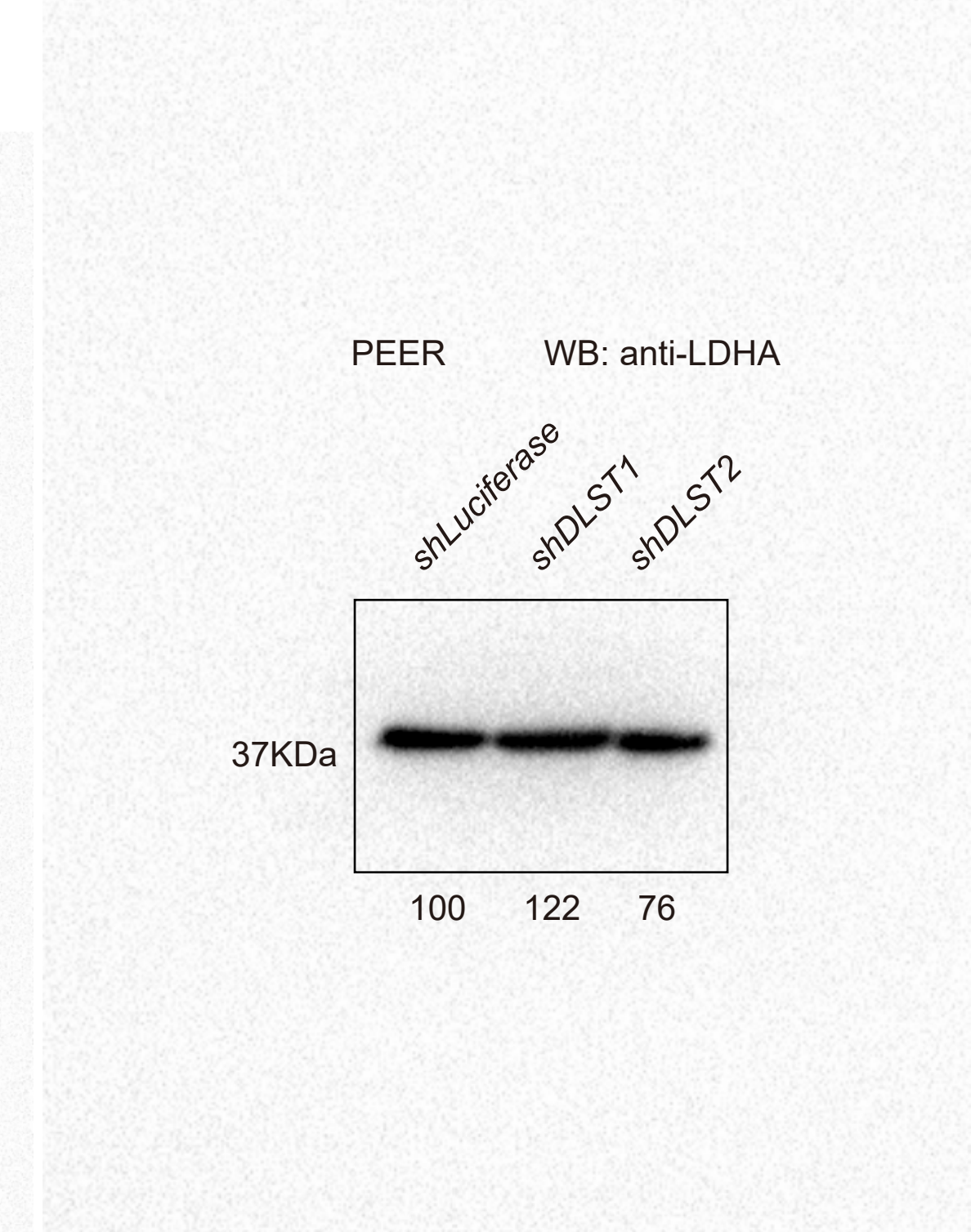

Supplement: Supplementary file 1 [file cancers-14-02983-s001.zip › Figure S1/Fig2 peer.pdf]

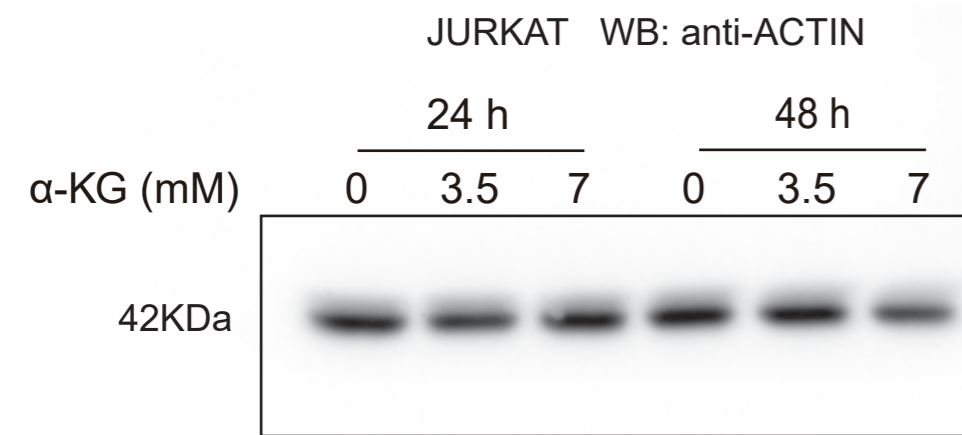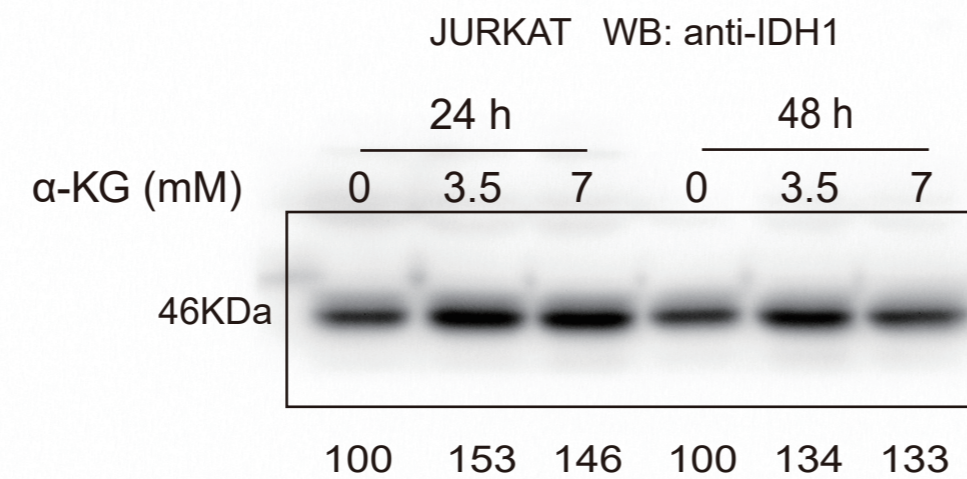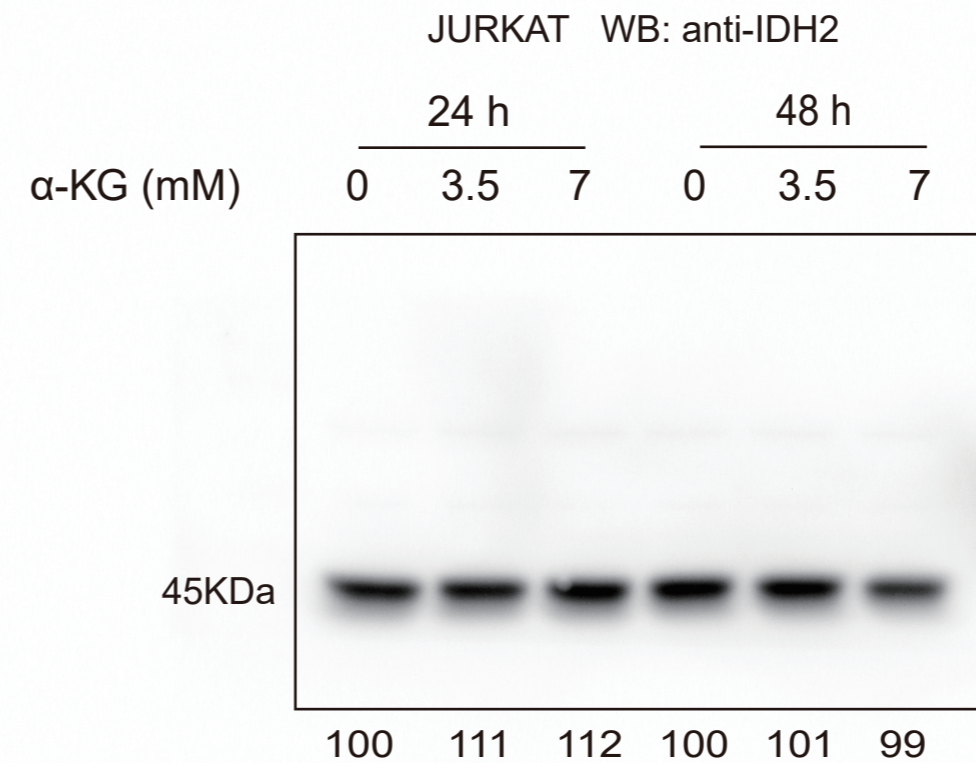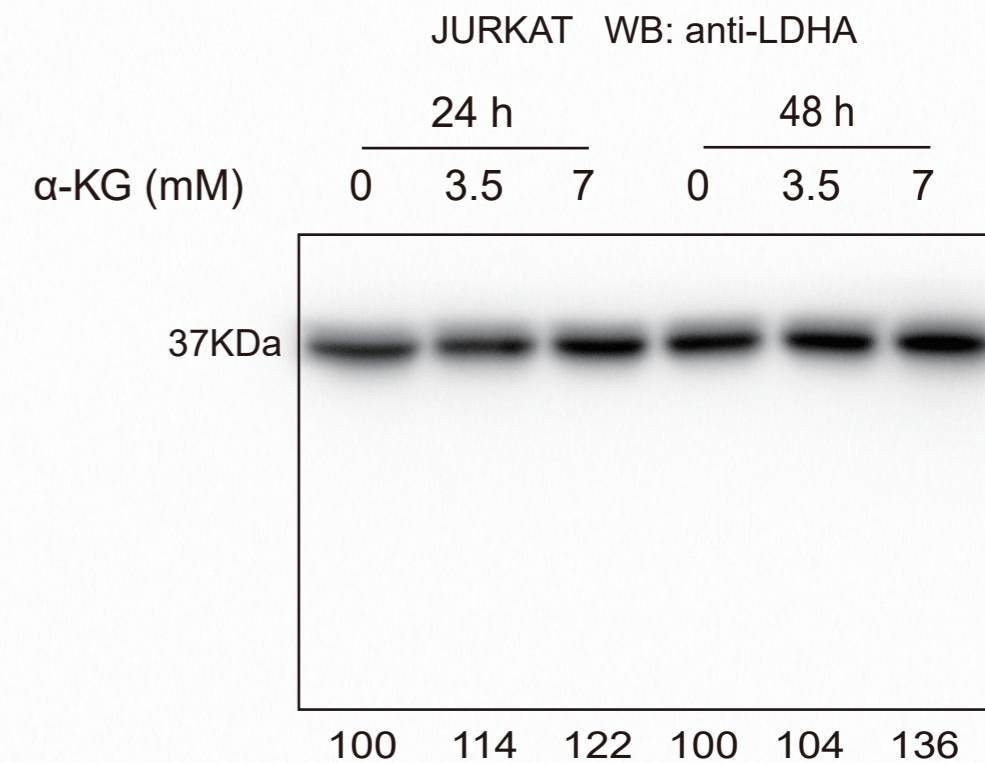

Supplement: Supplementary file 1 [file cancers-14-02983-s001.zip › Figure S2/Fig3 jurkat.pdf]

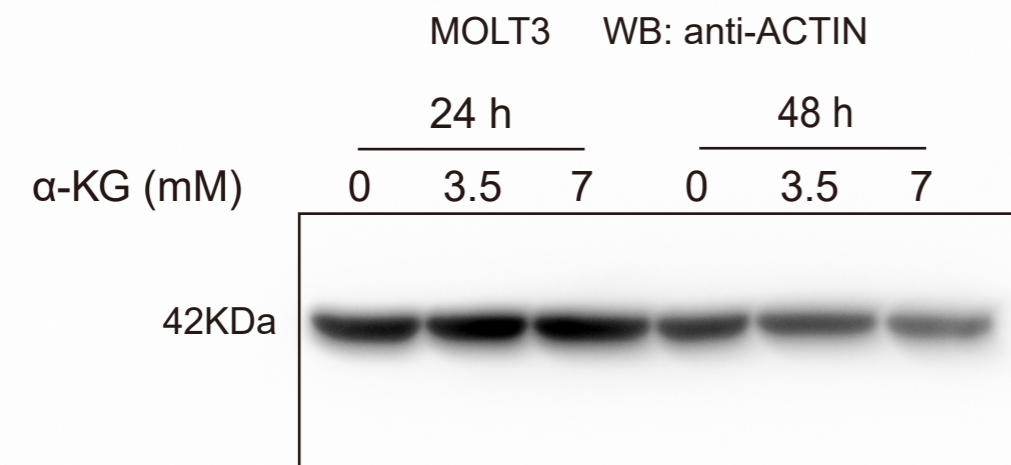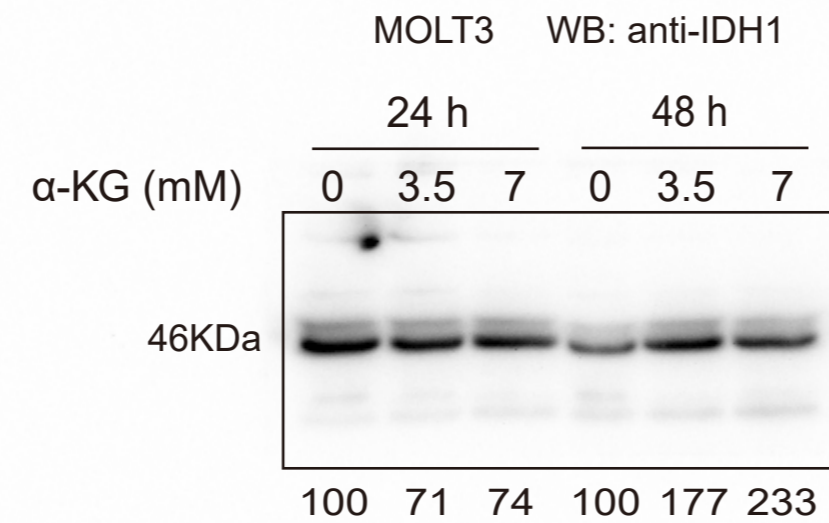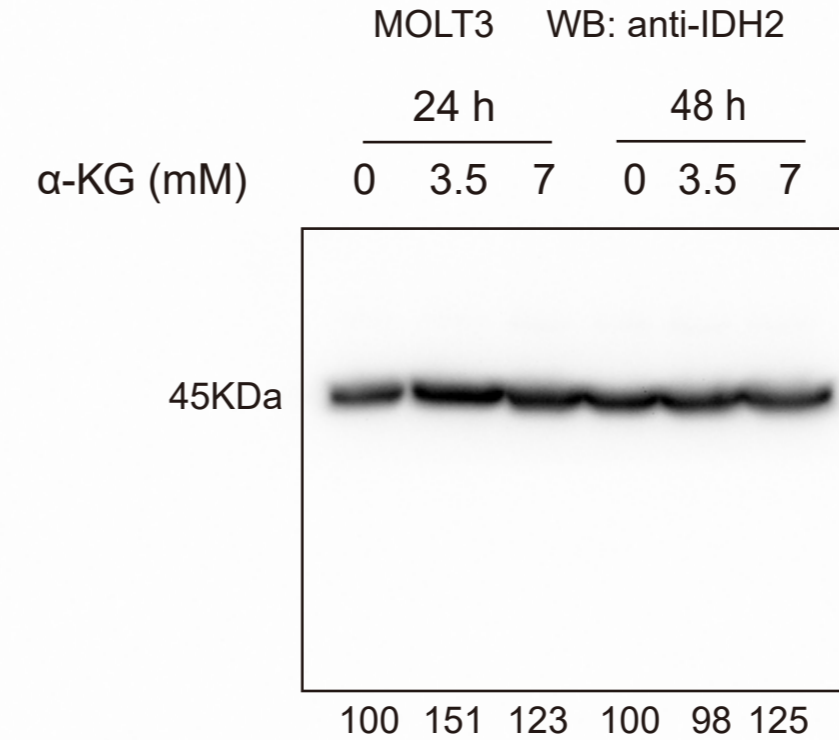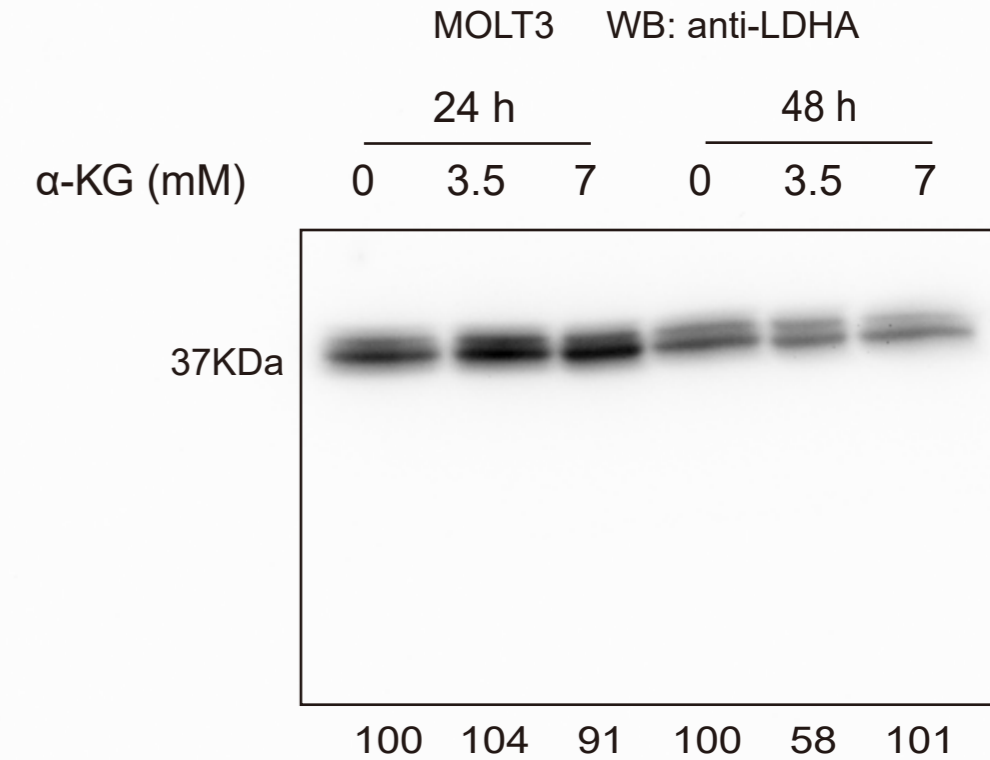

Supplement: Supplementary file 1 [file cancers-14-02983-s001.zip › Figure S2/Fig3 molt3.pdf]

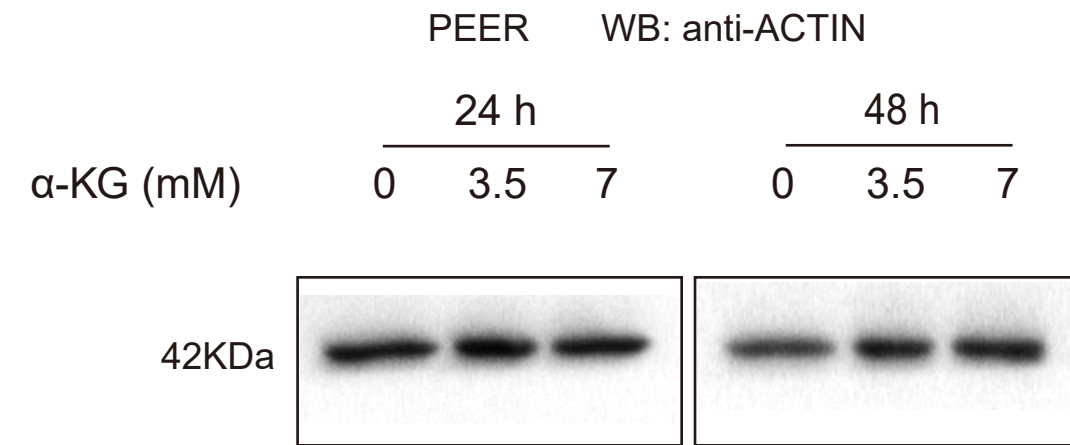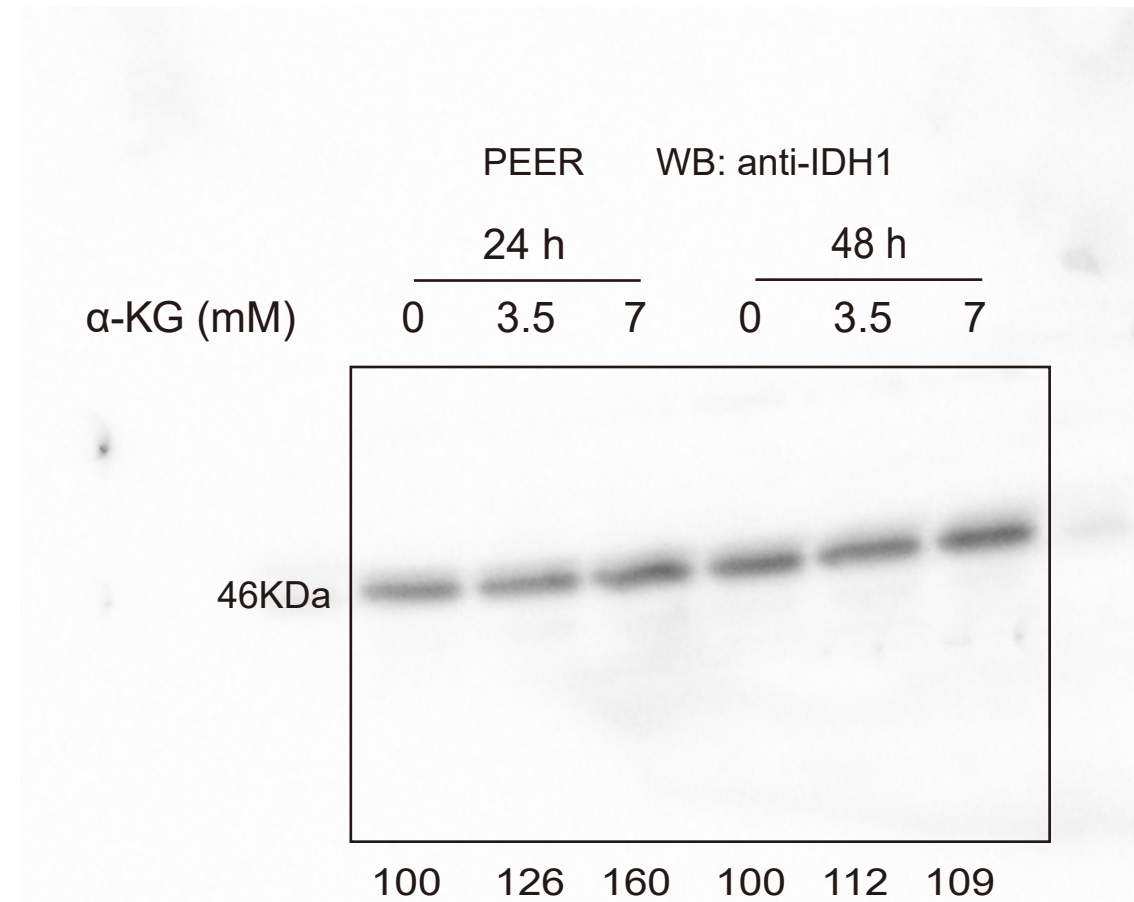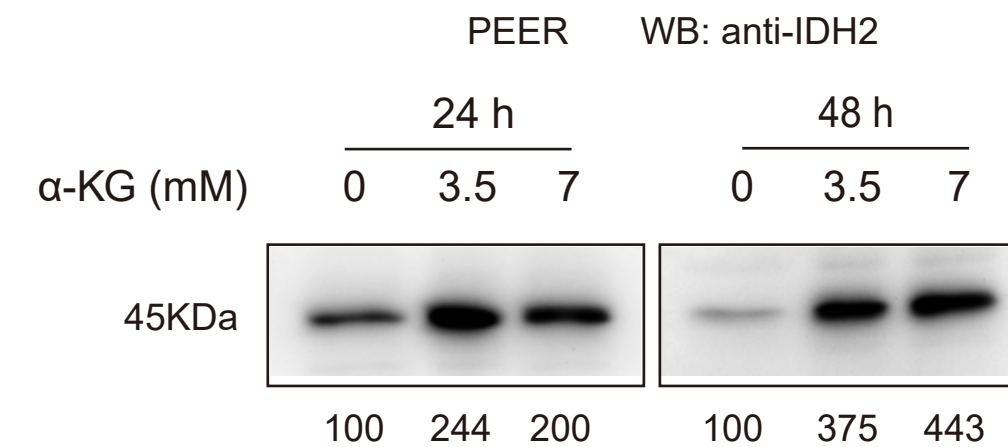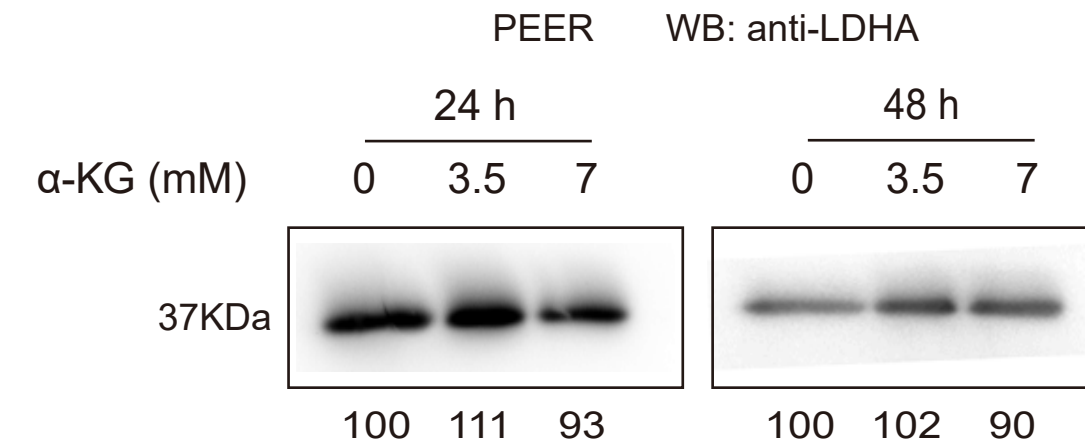

Supplement: Supplementary file 1 [file cancers-14-02983-s001.zip › Figure S2/Fig3 peer.pdf]
